# Hints to the People on the Profession of Medicine

**Published:** 1852-01

**Authors:** Wm. Maxwell Wood


*Hints to the People on the Profession of Medicine.* By Wm. Maxwell Wood, M. D.

The first edition of this little work, which has been tastefully gotten up by G. H. Derby & Co., is already exhausted. We are gratified to learn that there is quite a demand for it, and that the prospect is it will be extensively circulated. It cannot fail to exert a favorable influence in behalf of the profession of medicine. Physicians will find it an useful book to distribute among their acquaintances.

